# Identification of Key Gene Signatures Associated With Bone Metastasis in Castration-Resistant Prostate Cancer Using Co-Expression Analysis

**DOI:** 10.3389/fonc.2020.571524

**Published:** 2021-02-02

**Authors:** Zhongxiang Yu, Hanlin Zou, Huihao Wang, Qi Li, Dong Yu

**Affiliations:** ^1^ Department of Orthopaedics, Shuguang Hospital Affiliated to Shanghai Traditional Chinese Medical University, Shanghai, China; ^2^ Department of Orthopedics, Putuo Hospital Affiliated to Shanghai Traditional Chinese Medical University, Shanghai, China; ^3^ Shi’s Center of Orthopedics and Traumatology, Shuguang Hospital Affiliated to Shanghai Traditional Chinese Medical University, Shanghai, China; ^4^ Department of Oncology, Shuguang Hospital Affiliated to Shanghai Traditional Chinese Medical University, Shanghai, China; ^5^ Center for Translational Medicine, Second Military Medical University, Shanghai, China

**Keywords:** bone metastatic CRPC, differentially expressed genes, weighted gene co-expression network analysis, module, hub genes

## Abstract

About 80–90% of castration-resistant prostate cancer (CRPC) patients would develop bone metastasis. However, the molecular mechanisms of bone metastasis are still not clear. This study aimed to detect the differences between the tumor and normal samples in bone after metastatic colonization. Four transcriptional datasets (GSE32269, GSE101607, GSE29650, and GSE74685) were obtained from the GEO database. 1983 differentially expressed genes (DEGs) were first identified between tumor and normal marrow samples in GSE32269. Most of the top 10 up-regulated DEGs are related with prostate cancer, and the top 10 down-regulated DEGs are mainly related with bone development. Seven co-expression modules were then detected based on the 1469 DEGs shared by the four datasets. Three of them were found highly preserved among the four datasets. Enrichment analysis showed that the three modules were respectively enriched in Cell adhesion molecules (CAMs), Leukocyte transendothelial migration and cell cycle, which might play significantly important roles in the tumor development in bone marrow. Ten, 17, and 99 hub genes for each module were then identified. And four genes (C3AR1, IL10RA, LY86, and MS4A6A) were detect to be tightly related to progression of bone metastatic CRPC. ROC curve was plotted and AUC was calculated to distinguish tumor and normal bone marrow samples as well as bone and non-bone metastatic CRPCs. The present study identified key genes and modules involved in bone metastatic CRPCs, which may provide new insights and biomarkers for understanding of the molecular mechanisms of bone metastatic CRPC.

## Introduction

Prostate cancer (PCa) is one of the most common cancers and the tenth most common cause of cancers related mortality in men in China (1). The rankings rise first in men in the developed countries (2). Castration-resistant prostate cancer (CRPC) is an advanced form of prostate cancer by disease progression following surgical or pharmaceutical castration. This process is not inevitable, which is usually companied by poor prognosis and reduced survival time. To be known, CRPC patients are also at high risk of developing metastases. The common sites are bone, lymph nodes, liver, lungs and brain. Bone is the most prominent site for metastases. About 80–90% of CRPC patients develop bone metastases (3). Bone metastases could lead to the disorder of bone metabolism and induce skeletal related events (SREs), such as pathological fracture, spinal cord compression and hypercalcemia, which not only reduce survival time and life quality, but also increase burden of treatment (4).

However, the molecular mechanisms of bone metastases are still not clear. A widely accepted mechanism is the ‘seed and soil hypothesis’, which describes an interaction between circulating tumor cell and microenvironment of bone tissue (5). The communication between bone and cancer cells is believed to be critical for the development and progression of bone metastases (6, 7). Most of researches focus on dissecting the process of initiation to development of distant metastasis, such as cancer cells migrating through the endothelial cells to gain access to systemic circulation *via* the tortuous and leaky tumor vasculature and cell signaling aberrations (8, 9). A set of marked differences were identified between metastases and primary tumors and the subgroups of bone metastasis were also detected by transcriptome or proteome analysis (10–12). In addition, David A. Quigley et al. explore the genomic hallmarks and structural variation in metastatic PC, including bone metastatic CRPCs (13). However, these researches do not pay more attention on the state of tumor cells after metastatic colonization and also do not explore the differences between the tumor cells and normal cells in bone. This study aimed to identify the differences between tumor and normal bone marrow samples through differential expression analysis and weighted gene co-expression analysis. The identified key genes and modules will provide new insights for understanding of the molecular mechanisms and clinical treatment for bone metastatic CRPC.

## Methods

### Data Collection and Preprocessing

Four expression profile datasets containing CRPC bone metastasis were downloaded from the GEO database (https://www.ncbi.nlm.nih.gov/geo). Dataset GSE32269 was chosen for further analysis with 29 CRPC bone metastatic marrow samples and four normal bone marrow samples, which was used for bone cancer significantly expressed genes selection and correlated modules detection. The other three datasets GSE101607, GSE29650, and GSE74685 were kept with only CRPC bone metastatic samples, which was used to validate and screen the truly significant and preserved bone cancer related modules. Detailed information of datasets was shown in **Table 1**.

**Table 1:** Datasets of gene expression profiles.

GEO accession	Platform	Probe number	Total sample number	CRPC bone metastasis sample number	Normal bone sample number
GSE32269	GPL96	22283	55	29	4
GSE101607	GPL10558	48107	60	40	0
GSE29650	GPL6947	49576	30	30	0
GSE74685	GPL15659	38695	149	20	0

Before the analysis, all the raw data were reprocessed. Probes were mapped to the gene symbols. Empty probes and probes mapping to multiple genes were both discarded according to each annotation platform. If there were multiple probes that mapped to the same gene symbol, their mean values were considered as the gene expression value. The reprocessed data was normalized by the limma (linear models for microarray data) package in R (14).

### Identification of Differentially Expressed Genes

The eBayes analysis was used to detect the differentially expressed genes (DEGs) between metastatic bone marrow samples and normal marrow samples in GSE32269 using limma package (14). The adjusted P-value <0.05 and |log-fold change|>1 were set as the threshold for DEGs screening.

### Enrichment Analysis

R package clusterProfiler (15) was used for the Enrichment analysis. False discovery rate (FDR) < 0.05 was set as the threshold for the identification of significant GO-Enrichment terms and Pathway-Enrichment terms.

### WGCNA Analysis

The co-expression network analysis was performed using weighted gene co-expression network analysis (WGCNA) (16). First, the soft threshold for network construction was selected, which is the lowest power for which the scale-free topology fit index curve flattens out upon reaching a high value. Second, the function blockwiseModules was used for one-step network construction and module detection. The module eigengene (ME) of each module and the correlation between MEs was then calculated. Thirdly, module preservation was calculated between GSE32269 and the other three datasets using the function modulePreservation (17). The comparability of two datasets is assessed by correlating measures of average gene expression and overall connectivity of two datasets. The higher the correlations of these properties, the better chance you will have of finding similarities between the two datasets at subsequent stages of analysis. Fourthly, the key node (hub gene) was determined by high intramodule connectivity of genes. The cut-off criteria was set |cor.geneModuleMembership| > 0.8. According to the intramodule connectivity, the detected hub genes were visualized using VisANT software (18). Finally, the study (19) containing mRNA and clinical data of 444 metastatic CRPC samples was used to validate the hub genes and subjected to survival analysis. The database GEPIA2 containing TCGA datasets (20) and the database Oncomine containing cancer microarray datasets (21) were used to validate the expression levels of hub genes.

## Results

### DEG Identification for CRPC Bone Metastatic Patients

**Figure 1:**
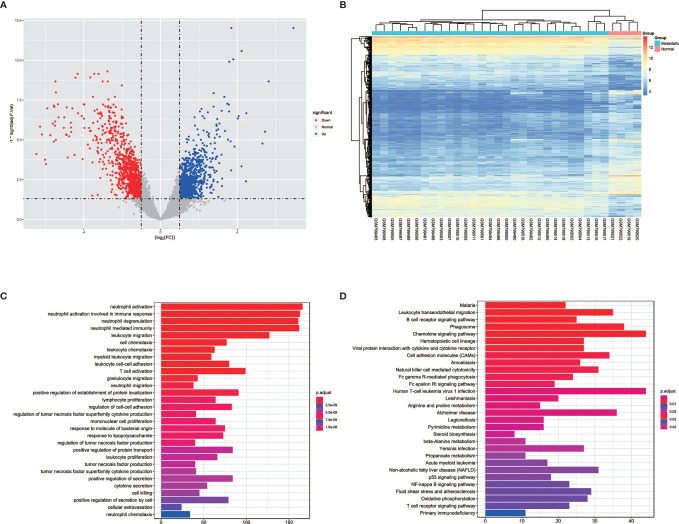
The volcano, heatmap, GO and KEGG enrichment results of differentially expressed genes (DEGs) between tumor and normal cells in bone. **(A)** The volcano plot for DEGs. Grey dots represent genes which are not differentially expressed, red dots represent the upregulated genes, and the blue dots represent the downregulated genes. **(B)** The heatmap for DEGs. **(C)** The annotation of gene ontology function of DEGs using GO enrichment analysis. **(D)** The annotation of pathway function of DEGs using KEGG enrichment analysis.

In order to detect the transcriptomic differences between CRPC bone metastatic marrow samples and normal marrow samples, the dataset GSE32269 with 29 CRPC bone metastatic marrow samples and four normal bone marrow samples was selected and downloaded from GEO databases. DEGs were identified using the limma package. 1983 DEGs were screened with the threshold of |logFC|>1 and p.adjust<0.05, as shown in >>**Figure 1A**, which contains 825 up-regulated genes and 1158 down-regulated genes for bone metastatic marrow samples (see **Supplementary Table 1**). The top 10 significantly expressed genes are KLK3, KRT18, EFNA1, SLC396A, NKX3-1, PGLYRP1, MGAM, RHD, GFI1, FAR2, of which the first five are up-regulated and the following five are down-regulated. The expression profiles of these DEGs were showed as a heatmap in **Figure 1B**. Enrichment analysis was further conducted. The result was shown in **Figures 1C, D**. The most enriched GO terms are neutrophil and leukocyte-associated terms. The top5 pathway terms are Malaria, Leukocyte transendothelial migration, B cell receptor signaling pathway, phagosome and chemokine signaling pathway.

### WGCNA Analysis

**Figure 2:**
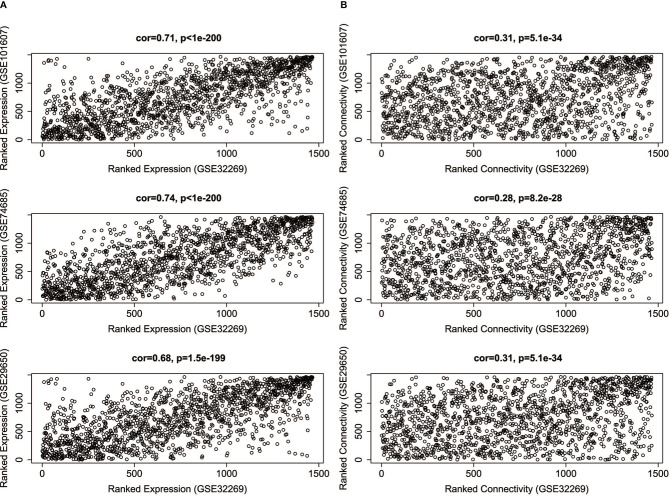
The correlations of average gene expression **(A)** and overall connectivity **(B)** between GSE32269 and other three datasets (GSE101607, GSE74685, GSE29650).

Since the four datasets come from different platforms, we should ensure that the four datasets are comparable. First, we need to limit the analysis to genes that expressed among the datasets. The intersection was taken among the DEGs of GSE32269 and the genes of other three datasets. 1469 genes were selected, and the corresponding expression profiles of these genes in four datasets were then prepared. Second, the comparability of GSE32269 and other dataset was assessed by measuring the average gene expression and overall connectivity between two datasets (**Figure 2**). It’s clear to see that the correlations are positive and the p-value are significant in all cases, which suggests that the datasets are comparable.

**Figure 3:**
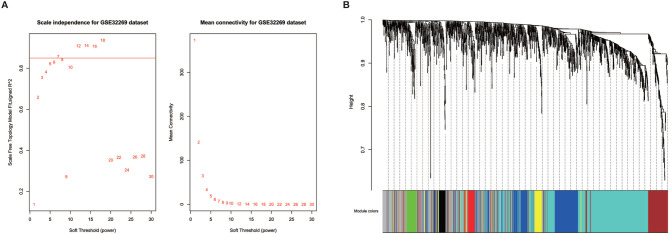
Identification of modules in the dataset GSE32269. **(A)** Network topology of different soft-thresholding powers. The left panel displays the influence of soft-thresholding power (x-axis) on scale-free fit index (y-axis). The right panel shows the influence of soft-thresholding power (x-axis) on the mean connectivity (degree, y-axis). **(B)** Clustering dendrogram showing eight modules that contain a group of highly connected genes. Each designated color represents a certain gene module.

Prior to gene co-expression network detection, the analysis of network topology for various soft-thresholding powers was performed to obtain relative balanced scale independence and mean connectivity. As shown in **Figure 3A**, power seven was the lowest power for which the scale-free topology fit index reaches 0.85. Based on this power, seven modules were generated as shown in **Figure 3B**. The largest module was the turquoise module, which contained 585 genes, the smallest module was the black module containing 49 genes. Averagely, each module contained 183 genes.

Enrichment analysis was further performed to detect biological significance of each module as listed in **Supplementary Table 2**. In the top 5 terms of each module, Yellow, Turquoise and Brown module were mainly enriched in neutrophil-associated GO terms, which were all related with leukocyte mediated immunity. Red module had no significantly enriched pathways. Yellow and brown module shared an enriched pathway term, named Osteoclast differentiation, which is related with bone development. It’s worth noting that turquoise enriched pathways contain a set of signaling pathways, such as B cell receptor signaling pathway, chemokine signaling pathway, NF-kappa B signaling pathway, Fc epsilon RI signaling pathway and hematopoietic cell lineage. These are reported to be related with tumorigenesis. In the yellow module enriched pathways, Cell adhesion molecules are related with cancer invasion and metastasis. The green module is enriched with cell cycle-associated pathways.

### Module Validation Among the Other Three Datasets

**Figure 4:**
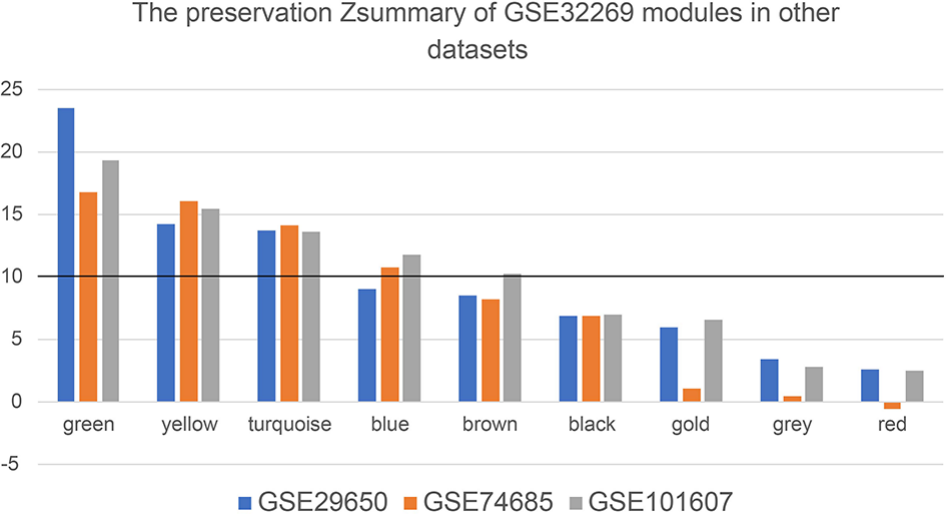
The preservation Zsummary values of eight GSE32269 modules in other datasets (GSE29650, GSE74685 and GSE101607). The black horizontal line is the threshold to define the highly preserved modules among the four datasets.

In order to detect whether these modules are preserved between the other three datasets, module preservation statistics were calculated using the function modulePreservation. The preservation Z-summaries was showed in **Figure 4**. We set the threshold Z>10 to screen the highly preserved modules. 3, 4, and 5 modules are separately found to be preserved in the dataset GSE29650, GSE74685 and GSE101607. And three modules (green, yellow, and turquoise) are shared and highly preserved in the three datasets, which were chosen for subsequent analysis.

### Identification and Validation of Hub Genes

**Figure 5:**
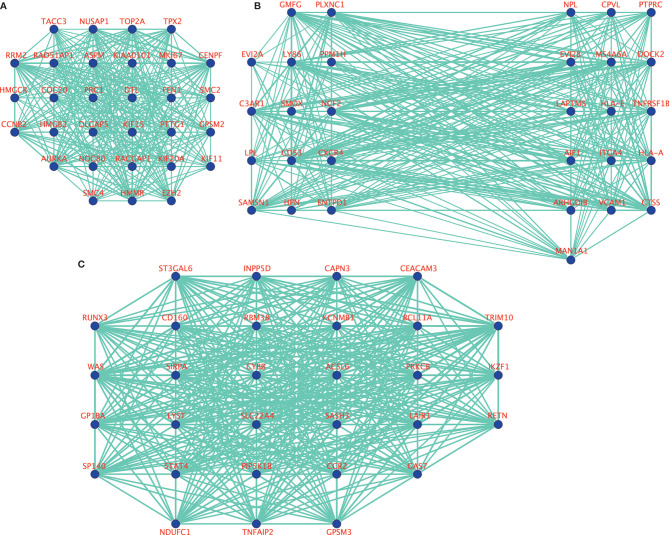
The visualization of hub genes in green module **(A)**, yellow module **(B)**, and turquoise module **(C)**.

**Figure 6:**
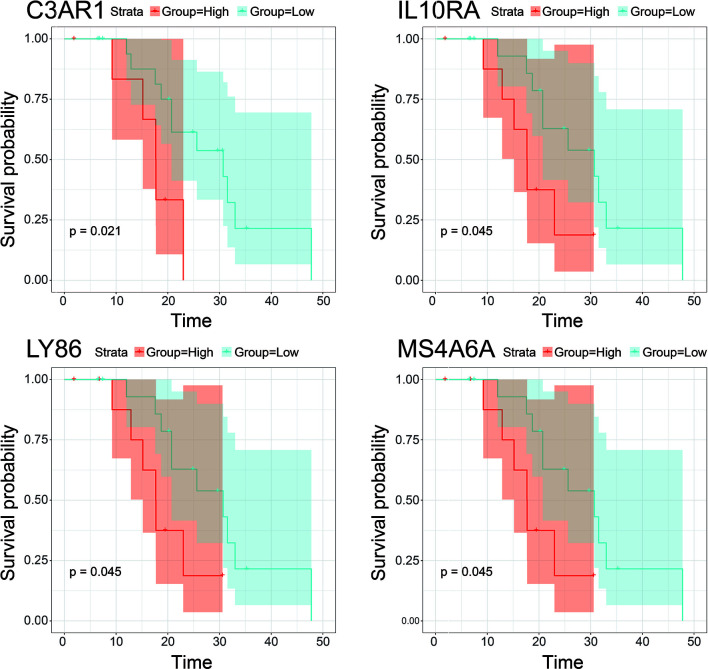
Survival analysis of hub gens with statistical significance (pvalue<0.05) in the dataset derived from Abida W’s study. Orange lines represent high expression of the hub genes and blue lines represent low expression.

**Figure 7:**
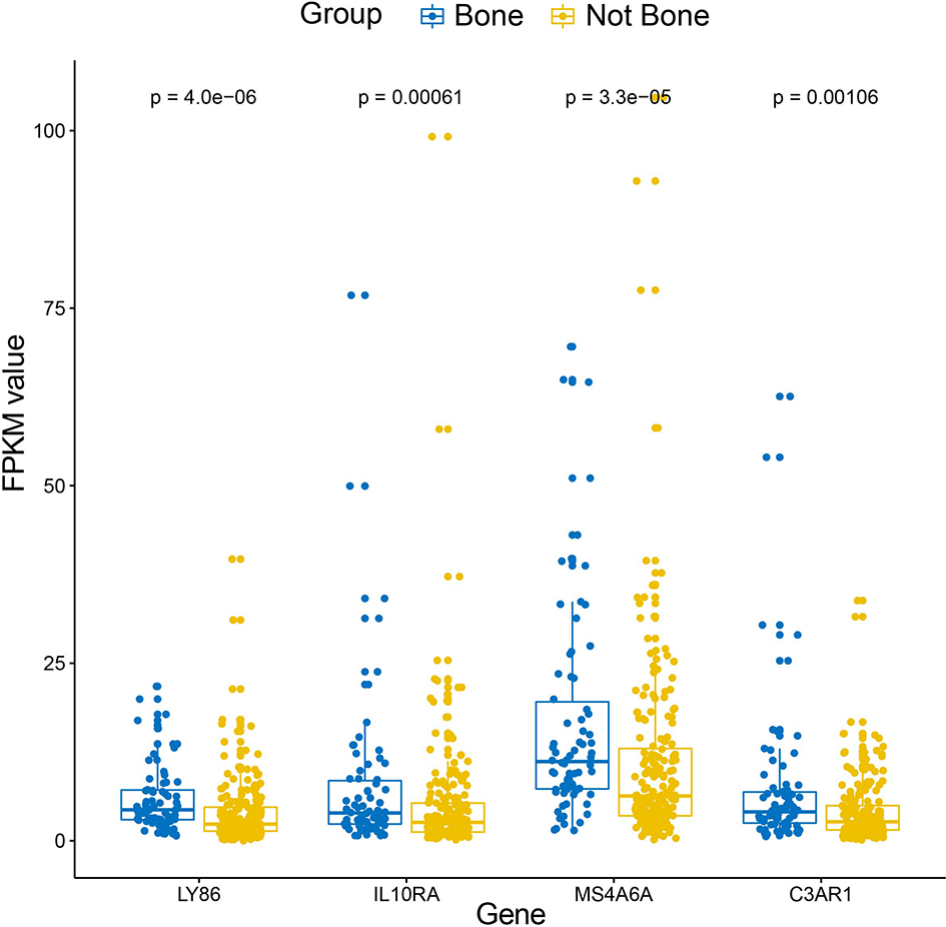
Box-plot of expression values (FPKM) of the four hub genes between bone and non-bone tissues in the metastatic CRPC patients derived from Abida W’s study.

10, 17 and 99 hub genes were separately identified in the three preserved modules (Green, Yellow, Turquoise). The corresponding networks of hub genes were showed in **Figure 5**. The study containing mRNA data followed clinical information of 160 bone metastatic CRPC samples and 284 non-bone metastatic CRPC samples were subjected to survival analysis and regression analysis. Four hub genes (C3AR1, IL10RA, LY86, and MS4A6A) were identified to significantly associated with the overall survival (**Figure 6**). The patients with lower expression of the genes had a longer survival. However, the four genes have significantly higher expression level in bone compared to other non-bone metastatic tissues as showed in **Figure 7**. In CRPC patients with metastases, the bone metastases have the worst median progression to non-bone tissues metastases (22).

**Figure 8:**
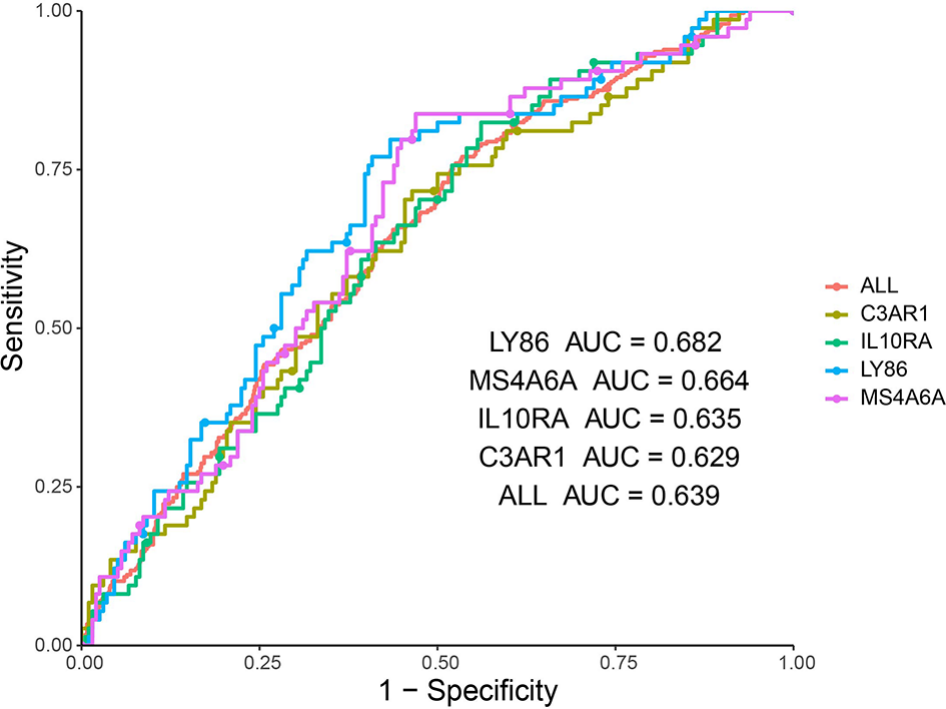
ROC analysis of four hub genes in the dataset derived from Abida W’s study. Receiver operating characteristic (ROC) curves and area under the curve (AUC) statistics is to evaluate the capacity of distinguishing bone and non-bone metastatic tissues in the metastatic CRPC patients.

In addition, ROC curve analysis was implemented to evaluate the capacity of the hub genes to distinguish bone and non-bone metastatic tissues. AUC values for the four genes were greater than 0.6 (**Figure 8**).

## Discussion

Development of bone metastases is a key and usual event in the progression of CRPC, which could lead to disorders of bone metabolism and skeletal related events. The median survival form men with bone metastases CRPC is approximately 1.5–2 years. The purpose of this study was to dissect the expression profile differences between the established metastatic tumor and normal bone marrow samples and then identified some key gene signatures and modules based on co-expression network analysis. These results will be helpful to deeply understand the molecular mechanisms of bone metastases and also provide candidate biomarkers for the prognosis prediction of bone metastatic CRPC patients.

The screened DEGs are found to be mainly related with prostate cancer and bone development. For example, among the top 10 up-regulated genes, KLK3 and KLK2, are highly enriched in prostate cancer, which are taken as effective biomarkers for diagnose and prognostic monitoring of prostate cancer (23). GOLM1 (24), FOLH1B (25), STEAP1 (26) and PLPP1 (27) are also identified as a candidate biomarker for prostate cancer. AGR2 expresses strongly in prostate tissue and show increased expression in prostate cancer (28). In a word, the up-regulated genes are mainly related with the tumorigenesis of prostate cancer. As for the top10 down-regulated genes, all of them are identified to be overexpressed in whole blood according to GTEx (29) and take part in embryonic development of blood and bone according to LifeMap Discovery (30). Therefore, the down-regulation of these genes would have effects on the function of bone or bone marrow, which might be genetic causes of SKE. These results indicated that the colonization in bone of metastatic CRPC cells not only keep the expression features of prostate cancer, but also induce new expression variations associated with bone. In another way, these results suggest the tissue specificity of DEGs, and the reliability of our results. Therefore, it is important to further dissect the expression differences between the established tumor and the normal bone marrow samples.

After a series of bioinformatic analysis, four hub genes identified from the three highly preserved co-expression modules among the four datasets were found to be tightly associated with overall survival in bone metastases CRPC patients. At present, there are no direct evidences to verify the functions of the four genes in prostate cancer or bone metastatic CRPC, but a set of researches showed that these genes were involved in the tumorigenesis and tumor proliferation in other cancers. It was shown that C3AR1 was significantly correlated with the overall survival In glioblastoma, which showed a longer survival time in the patients with lower expression of C3AR1 (31). In a recent study, over-expression of C3AR1 was proved to promotes HL-60 cell migration and invasion *in vitro* experiment (32). In other words, down-expression might decrease the migration and invasion capacities of tumor cells. Moreover, C3a, which binds to an orphan G protein-coupled receptor encoded by C3AR1, was reported as an immune regulator in the tumor microenvironment and act as insidious propagators of tumor growth and progression (33). In this respect, the down-regulation of C3AR1 might inhibit the process of tumor growth and progression. Therefore, these may be the reasons why the patients with lower expression of C3AR1 had good prognosis. IL10RA encodes a receptor for interleukin 10, which can inhibit the synthesis of proinflammatory cytokines. In colorectal cancer, the expression of IL10RA is found to be higher in healthy tissue than in the CRC tissue and showed association with the proliferation index, confirming the importance of IL10RA in the pathogenesis of CRC (34). However, increased level of IL10RA in the study population was not linked with overall survival time. In diffuse large B-cell lymphoma, IL10 receptor is highly expressed and predicts worse survival (35). Functional experiment showed that IL10 receptor plays an important role in IL10-JAK-STAT signaling pathway. Blocking IL10R would interrupt the IL10 autostimulatory loop and lead to cell death through cell cycle arrest and introduction of apoptosis. LY86 encodes the lymphocyte antigen 86, which may cooperate with CD180 and TLR4 to mediate the innate immune response to bacterial lipopolysaccharide (LPS) and cytokine production. LY86 was identified as a novel biomarker for the prediction of osteosarcoma prognosis and therapeutic targets (36). Moreover, healthy hematopoietic stem progenitor cells (HSPCs) can be transformed genetically by leukemia macrovesicles to over express LSC specific genes, which contains LY86, LRG1 and PDE9A and so on (37). These suggests that LY86 might play an important role in the transformation of localized normal bone marrow cells to cancer cells. MS4A6A encodes a member of the membrane-spanning 4A gene family, which display unique expression patterns among hematopoietic cells and nonlymphoid tissues. GWAS researches showed that MS4A6A is associated with heel bone mineral density and Alzheimer’s disease (38). MS4A6A was reported to be highly expressed in putative Tumor-associated macrophages (TAMs) populations. Previous reports suggest that TAMs may show an immunosuppressive M2 signature, which promotes tumorigenesis by suppressing immune surveillance and inducing angiogenesis, rather that the activating M1-type signature (39). In addition, a recent study found that high expression of MS4A6A was associated with poor progression-free survival of ovarian cancer (40), which is consistent with the result of this study. Therefore, this gene might take an important role in the colonization of metastatic cancer cells in bone marrow and tumorigenesis of localized bone marrow cells. In above-mentioned studies, high expression of the four genes are all significantly associated with poor prognosis, which is consistent with the performances. These will serve as important references to explore the molecular mechanisms of the genes on bone metastatic CRPC.

**Figure 9:**
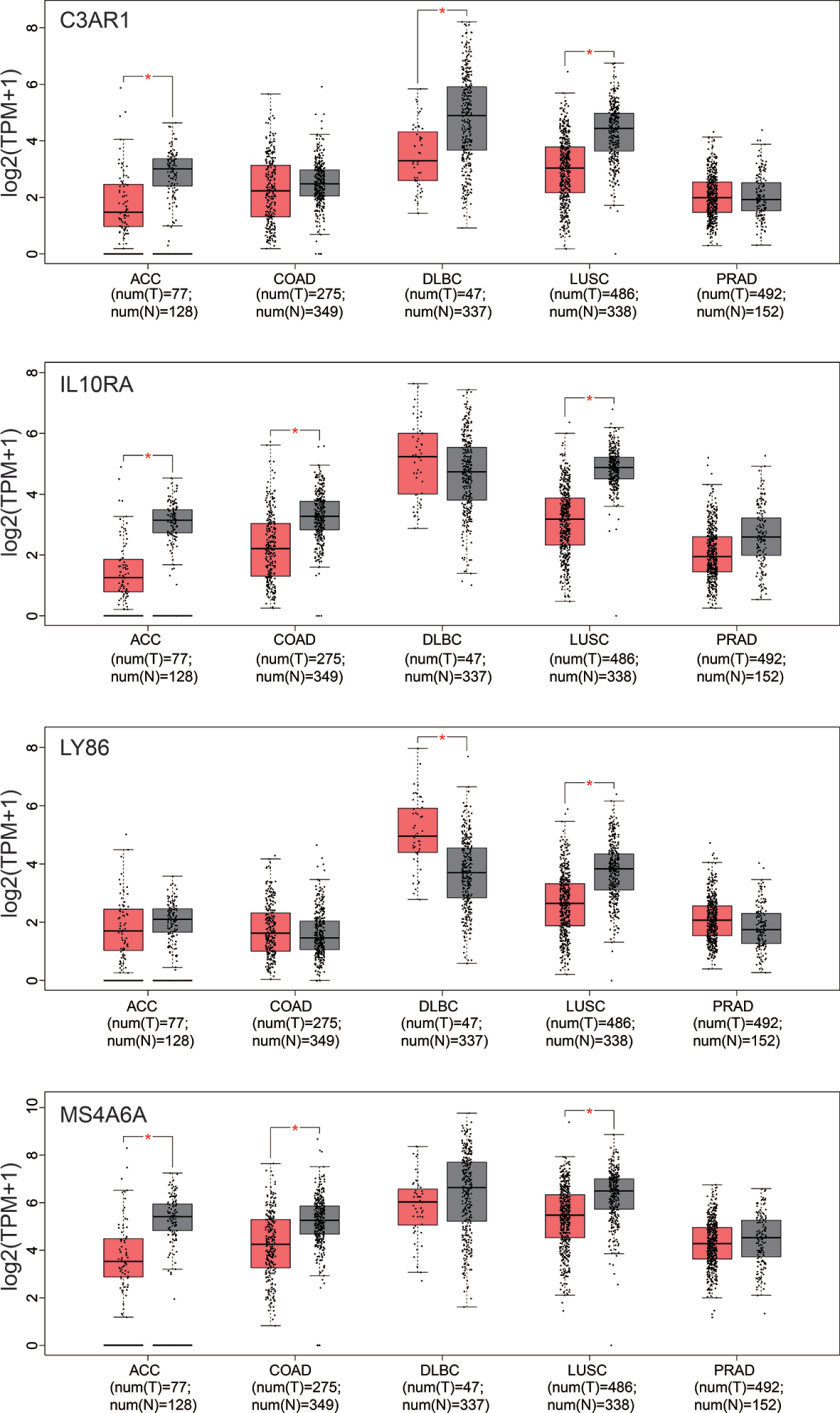
Box-plot of expression values (TMP) of the four hub genes between tumor and normal samples derived from the TCGA datasets. ACC, Adrenocortical carcinoma; COAD, Colon adenocarcinoma; DLBC, Lymphoid Neoplasm Diffuse Large B-cell Lymphoma; LUSC, Lung squamous cell carcinoma; PRAD, Prostate adenocarcinoma. Differential analysis between tumor and normal group was conducted using one-way ANOVA method. *pvalue < 0.05.

**Figure 10:**
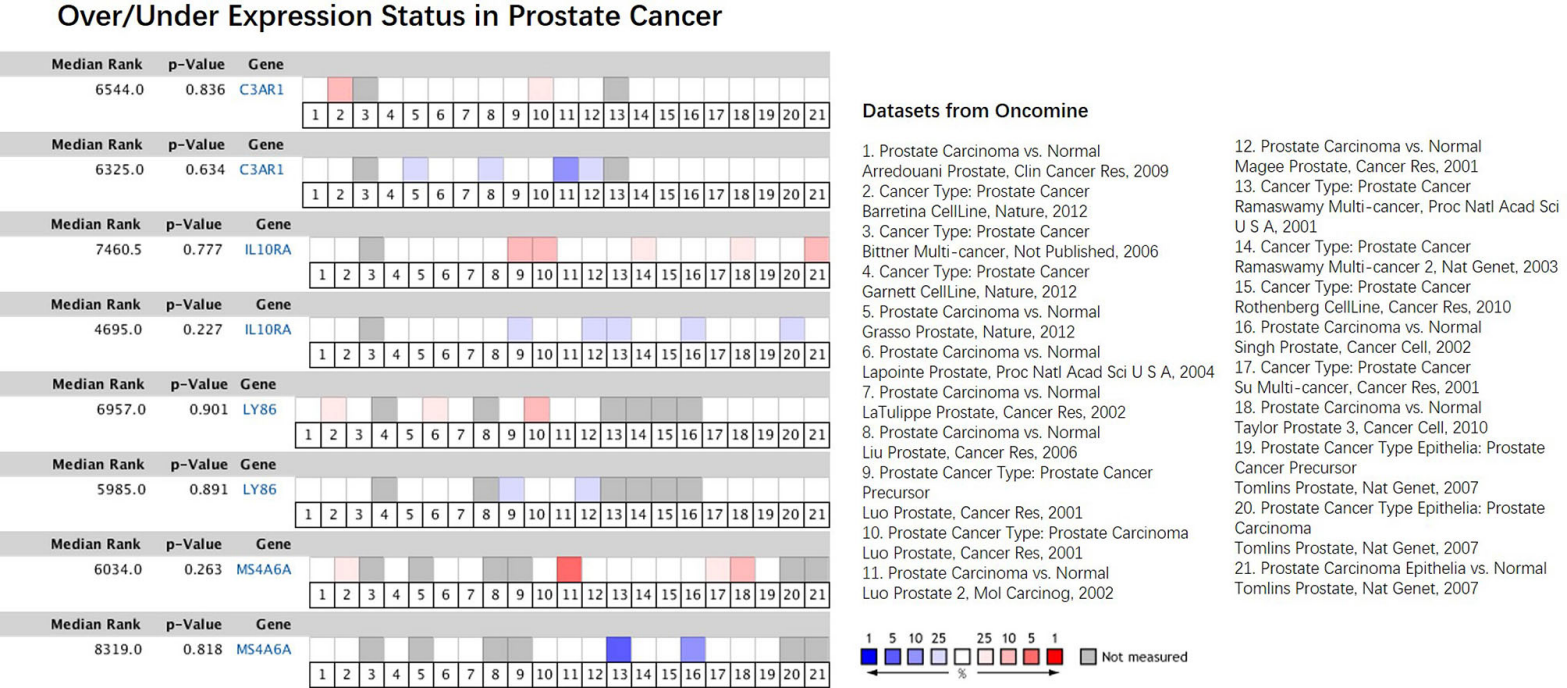
Comparison of the four hub genes across 21 analyses of prostate cancer datasets. The colored box denotes the gene’s percentile rank for that analyses. The more saturated the color, the higher the percentile rank. Red denotes over-expression; blue denotes under-expression. The datasets were listed on the right.

In our results, the four genes were all down-regulated in tumor bone marrow samples compared to the normal samples, which was different from the performances in other tumors described above, However, the four genes present consistency trends as this study in the lung squamous cell carcinoma according to TCGA datasets (20). Some of the four genes were also lowly expressed in ACC, COAD or DLBC (**Figure 9**). We also made a search of the four genes in Oncomine database (21) with parameters (Analysis Type: Differential analysis, Cancer vs. Normal analysis, Prostate cancer vs. Normal analysis; Data Type: mRNA). The results showed that these genes have no differences in expression between tumor and normal samples in most of prostate cancer datasets as listed in the **Figure 10**, which is consistent with the result in the TCGA prostate cancer dataset.

At present, a growing number of researches focus on the communication between tumor cells and bone stroma (41). Existing discoveries show that a vicious cycle of molecular crosstalk between tumor cells and the bone metastatic niche often take place in osteolytic bone metastasis (42). Targeting the bone metastatic niche is also evolving into a promising avenue for the prevention of bone metastatic relapse, therapeutic resistance, and other aspects of cancer progression (43–45). Therefore, it is meaningful and important to dissect the differences between tumor cells and bone metastatic niche at different level, including transcriptome, which will be essential to explore the molecular mechanisms or interaction underlying the bone metastases and new clinical practice. Based on this consideration, this study has creatively used public data to dissect the expression differences between established tumor and normal bone marrow samples derived CRPCs. The first screened DEGs were involved in prostate cancer and bone development. And the followed illustrated four hub genes are not only associated with overall survival of bone metastatic CRPC samples, but also be capable of distinguishing bone metastases and non-bone metastases. These findings would greatly provide new insights and biomarkers for understanding of the molecular mechanisms and clinical treatment for bone metastatic CRPC.

## Data Availability Statement

Publicly available datasets were analyzed in this study, these can be found in here: the NCBI Gene Expression Omnibus (GSE32269, GSE101607, GSE29650 and GSE74685).

## Author Contributions

ZY and DY designed the study and drafted the manuscript. ZY, HZ, and HW performed all the data analysis. HW helped the preparation of figures and tables. QL and DY contributed to the writing of the manuscript. All authors contributed to the article and approved the submitted version.

## Funding

This work was supported by Shanghai University of Traditional Chinese Medicine research grants (18LK038). The funders had no role in study design, data collection and analysis, decision to publish, or preparation of the manuscript.

## Conflict of Interest

The authors declare that the research was conducted in the absence of any commercial or financial relationships that could be construed as a potential conflict of interest.

## Supplementary Material

Supplementary Table 1 All the differentially expressed genes between metastatic tumor and normal bone marrow samples in GSE32269.

Supplementary Table 2 GO and KEGG enrichment analysis results for each module.

## Abbreviations

CRPC, Castration-Resistant Prostate Cancer; DEG, Differentially Expressed Gene, WGCNA, Weighted Gene Correlation Network Analysis; ME, module eigengene.
